# Uncovering the pathways underlying whole body regeneration in a chordate model, *Botrylloides leachi* using *de novo* transcriptome analysis

**DOI:** 10.1186/s12864-016-2435-6

**Published:** 2016-02-16

**Authors:** Lisa E Zondag, Kim Rutherford, Neil J. Gemmell, Megan J. Wilson

**Affiliations:** Department of Anatomy, Otago School of Medical Sciences, Developmental Biology and Genomics Laboratory, University of Otago, P.O. Box 56, Dunedin, 9054 New Zealand; Department of Anatomy, Centre for Reproduction and Genomics and Allan Wilson Centre for Molecular Ecology and Evolution, University of Otago, P.O. Box 913, Dunedin, 9054 New Zealand

**Keywords:** Whole body regeneration, *Botrylloides leachi*, Transcriptome

## Abstract

**Background:**

Regenerative capacity differs greatly between animals. In vertebrates regenerative abilities are highly limited and tissue or organ specific. However the closest related chordate to the vertebrate clade, *Botrylloides leachi*, can undergo whole body regeneration (WBR). Therefore, research on WBR in *B. leachi* has focused on pathways known to be important for regeneration in vertebrates. To obtain a comprehensive vision of this unique process we have carried out the first *de novo* transcriptome sequencing for multiple stages of WBR occurring in *B. leachi*. The identified changes in gene expression during *B. leachi* WBR offer novel insights into this remarkable ability to regenerate.

**Results:**

The transcriptome of *B. leachi* tissue undergoing WBR were analysed using differential gene expression, gene ontology and pathway analyses. We observed up-regulation in the expression of genes involved in wound healing and known developmental pathways including WNT, TGF-β and Notch, during the earliest stages of WBR. Later in WBR, the expression patterns in several pathways required for protein synthesis, biogenesis and the organisation of cellular components were up-regulated.

**Conclusions:**

While the genes expressed early on are characteristic of a necessary wound healing response to an otherwise lethal injury, the subsequent vast increase in protein synthesis conceivably sustains the reestablishment of the tissue complexity and body axis polarity within the regenerating zooid. We have, for the first time, provided a global overview of the genes and their corresponding pathways that are modulated during WBR in *B. leachi*.

**Electronic supplementary material:**

The online version of this article (doi:10.1186/s12864-016-2435-6) contains supplementary material, which is available to authorized users.

## Background

**Fig. 1:**
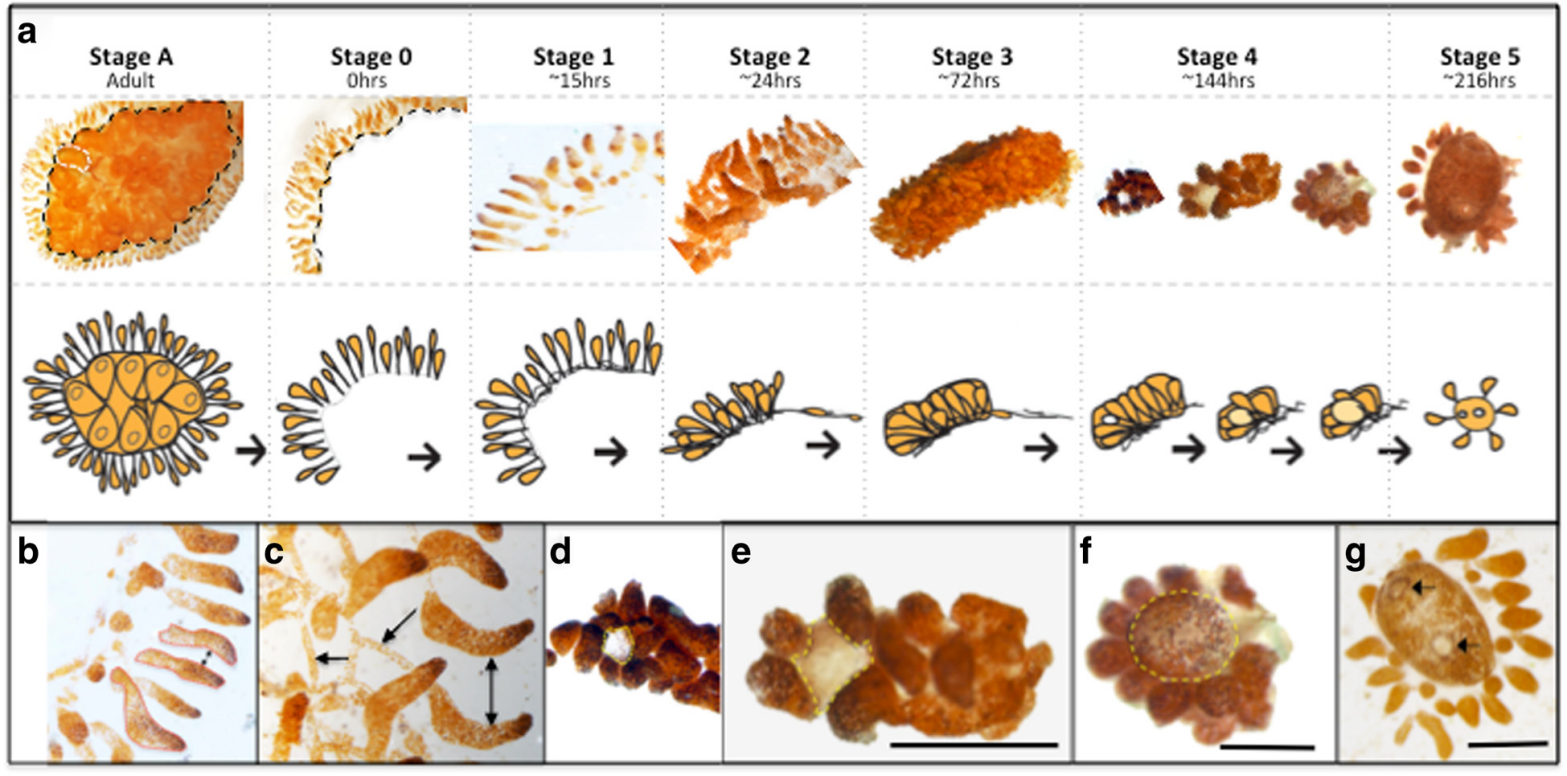
Staging scheme used for WBR in *B. leachi.* **a** Stage A: *B. leachi* colony prior to dissection. Black dashed line indicates the dissection sites. Stage 0: Marginal ampullae at 0 h, directly after dissection from the zooids. Black dashed line indicates dissection site. Stage 1: New vascular connections formed between ampullae, creating the beginning of a new circulatory system. Stage 2: Marginal ampullae starting to condense together, creating a compact network of blood vessels within the tunic matrix. Stage 3: Further condensing of the blood vessels. Stage 4: Formation of small transparent vesicle (regeneration niche) in the middle of the condensed blood vessels. The regeneration niche continues to expand in size, gaining pigmentation and ultimately forming the new adult. Stage 5: A fully developed zooid capable of filter feeding forms ~ 8 days. **b** Higher magnification image of the terminal ampullae at Stage 0. Red line surrounding individual ampullae and double arrow indicating space between two ampullae. This distance inversely correlates with the time it takes the vascular tissue to reach Stage 3. **c** Same as **b**, with arrows pointing at blood vessels connecting individual ampullae to one another. **d**–**f** Higher magnification images of Stage 4. Yellow line surrounding the regeneration niche that grows to form the new adult. **g** Adult zooid capable of filter feeding. *Arrows* indicating the two siphons present (atrial and peribranchial siphons). Scale bar represents 0.5 mm

*Botrylloides leachi* is a colonial tunicate found in shallow waters attached to rocks, pilings, floats and other submerged surfaces [1–3]. A single *B. leachi* colony is composed of numerous genetically identical zooids (adults) enveloped by an extracellular tunic comprised of a carbohydrate-derived gelatinous matrix [2, 4]. Individual zooids are approximately 2–3 mm in length and are organized into systems composed of two parallel rows connected through a fine blood vessel network (Fig. 1a, Stage A) [1, 3, 5]. As a chordate, *B. leachi* falls into the same phylogenetic grouping as vertebrate animals [6]. In general, regenerative capacity correlates inversely with tissue complexity, thus complex organisms such as chordates have a limited capability to regenerate following severe injury. A unique exception to this trend are colonial tunicates such as *B. leachi* that are capable of whole body regeneration (WBR); [1] where a fully functional adult organism is regenerated from a minuscule piece of vascular tissue (~200 cells), restoring both somatic and germ cell lines. Either loss of all adults or dissection of the ampullae and associated blood vessels from the zooids results in the regeneration of a new adult. This whole process occurs within 8–14 days and can be reproduced in the laboratory [3]. However, very little is known about the genetic regulation of *B. leachi* regeneration, partly due to the lack of genome and molecular data available for this species. Undoubtedly large-scale gene expression changes must be associated with the initiation, development and completion of WBR. To characterise these, we have used a *de novo* transcriptomic approach to gain a broader understanding of the mechanisms underpinning this unique regeneration phenomena.

It is hypothesized that pluripotent stem-like cells, potentially undifferentiated hemoblasts, are the likely progenitor cells for WBR and asexual reproduction [4, 5, 7, 8]. *B. leachi* regeneration is thus initiated through the activation of these dormant cells located attached on the surface of the vascular epithelium [2, 6, 8]. Upon activation, they migrate from the epithelium into the vascular lumen locally remodeled into regeneration niches. Here, these progenitor cells proliferate, differentiate and finally form a single new adult within a regeneration niche [3, 4, 7, 8]. A range of pluripotent cell lineage markers such as *piwi, pl10*, *raldh*, *vasa* and *SoxB1* are expressed in botryllid hemocytes suggesting that they are the source of progenitor cells required for asexual budding and regeneration [4, 8]. During WBR *piwi* positive cells also appear, initially lining the vasculature before mobilizing into the vascular lumen [8]. Notably, it still remains uncertain how these cells located within the vascular epithelium of *B. leachi* are induced to differentiate and undergo WBR.

Prior studies investigating *B. leachi* WBR have been limited to cloning fragments of candidate factors required for regeneration in other animals, and expressed-sequence tag (EST) screens [1, 3]. Here we have used RNA sequencing (RNA-seq) followed by *de novo* transcriptome assembly to characterize WBR in *B. leachi*. This approach enables differential expression analyses for species with little previous genetic characterization. RNA libraries were sequenced from six stages of regeneration, along with a library for a whole (intact) adult colony and a *B. leachi* embryo (larvae) stage. We first assembled a reference transcriptome, which was then used to explore the transcriptomic profiles of the individual libraries. These highly novel data sets were then compared using differential expression analysis to provide unprecedented insight into WBR.

## Results and discussion

### Inducing *B. leachi* regeneration

To analyse gene expression, we collected total RNA from regenerating colonies at key stages during this process. We artificially induced regeneration to occur in *B. leachi* by dissecting away the adults and leaving only the terminal ampullae. To accurately pool samples from the same regeneration stage together for RNA sequencing, we used both time and observable morphological change. For the first 24 h after adult removal time was a consistent and reliable tool for pooling, however beyond 24 h the progress of the regeneration process became highly variable so samples were pooled based upon visible changes (Fig. 1a). The removal of the zooids was followed by a brief hemorrhagic period, which was quickly (~1 min) stopped through vascular contraction (Fig. 1a; Stage 0 and Fig. 1c-d), thus preventing further loss of circulatory cells. Blood circulation resumed a normal bidirectional flow within the first 24 h, once new connection were formed between the existing vasculature (Fig. 1a; Stages 1–2). The tissue then underwent considerable reorganisation as it condenses together inside the tunic (Fig. 1a; Stage 3). During stages 2–3, vascular cells aggregate into regeneration niches, which become visible by stage 4 as a light opaque mass in the middle of the dense vascular tissue (Fig. 1a and d-f; Stage 4). This opaque cluster of cells grew in size over the next few days and developed into a complete functional adult sea squirt (Fig. 1a and g; Stages 5). Total RNA was collected from each of these five stages of regeneration (Fig. 1a; Stages 1–5), as well as isolated vascular tissue (Fig. 1a; Stage 0) and intact *B. leachi* colony (Fig. 1a; Stage A) for sequencing on an Illumina platform.

### *De novo* transcriptome assembly and quality analysis

A total of ~316 million reads from all eight sequenced stages were used for *de novo* transcriptome assembly and analysis. All the raw sequencing read have been submitted to NCBI Sequence Read Archive (SRA; http://www.ncbi.nlm.nih.gov/sra) under accession number SRP064769. Each of the RNA libraries had an average Q score of over 30 for more than 90 % of the sequencing reads (Additional file 1). To compensate for the absence of a reference genome for *B. leachi,* we first assembled a *de novo* transcriptome that we used as a reference to determine read counts for each stage of regeneration [9]. The *de novo* transcriptome was assembled using Trinity [10] and produced a total of 59,354 contigs, with an N50 of 2,229 bp and a total sequence length of 66,522,588 bp. Chimera percentage in the transcriptome was estimated using TransDecoder to be 6.94 % [10].

To estimate the completeness of the assembled reference transcriptome, the Core Eukaryotic Genes Mapping Approach (CEGMA) was used [11, 12]. CEGMA approximates the completeness of either a genome or transcriptome based on the presence/absence of 248 core eukaryotic genes (CEG). The greater the number of CEGs identified, the more complete the assembly. Out of the 248 core genes, 237 (95.6 %) were identified in the *B. leachi* transcriptome and marked as ‘complete’ (determined as per Parra, et al. 2007) (Additional file 2). An additional 7 ‘partial’ genes (2.8 %) of the CEG were also found.

To annotate the *B. leachi* reference transcriptome, all 59354 of the assembled transcripts were aligned using blastx [13] to the *C. intestinalis* (taxid:7719, Ensembl release #81, July 2015) protein database (Additional file 3). We chose the *C. intestinalis* genome over that of *Botryllus schlosseri* [14], a sister botryllid species, because its annotation was more complete at the time of analysis. We found that 23801 (i.e. 40.1 %) of the *B. leachi* transcripts had a significant match to the *C. intestinalis* protein database (using a cut-off at E-value < 1e-10, Additional file 3). In total, 17302 *C. intestinalis* proteins were identified, of which 11880 (68.7 %) were matched by at least one *B. leachi* transcriptome contig. When compared to the UniProtKB protein database [15], release 2014_09) 28295 (47.7 %) of the transcript sequences had a significant match (using a cut-off at E-value < 1e-10, Additional file 4).

**Fig. 2:**
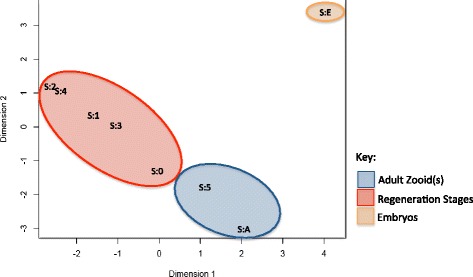
Multi-dimensional scale (MDS) plot generated for all RNA samples. The MDS plot shows clustering of samples based on the distance derived from biological coefficient of variation (BCV) between the paired samples most heterogeneous genes. The red oval indicates stages undergoing regeneration. Blue oval are the two stages with zooids included; Stage A whole colony and Stage 5 when a new adult has regenerated. The yellow oval indicates the embryonic staged RNA, with a very different MDS values than those that samples from regenerating or mature *B. leachi* colony

A multi-dimensional scaling plot (MDS) was constructed to examine the transcriptional similarity between samples before differential gene expression was carried out. The MDS plot shows clustering of samples based on the biological coefficient of variation (BCV) between paired samples [16] (Fig. 2). The more closely related the samples (less biological variation), the smaller distance between them when displayed in the MDS plot. Stages 2 and 4 were the closest pair on the MDS plot which implies that these two conditions are most similar out of four included in the study. Three independent clusters can be identified: first one spanning all the regeneration stages, a second cluster that includes RNA collected from the two stages with adult(s) present (Stages A and 5) and a third cluster for the embryonic stage (Stage E). RNA collected from the embryonic stage had a high BCV, indicating that gene expression in this samples was much more heterogeneous, when compared to both the regenerating and the adult samples. This suggests that the gene expression profile during embryonic development differs to that of blastogenic reproduction (intact colonies) and samples from colonies undergoing WBR.

### Differential gene expression analysis and validation with qRT-PCR

Following assembly of a reference transcriptome, we mapped read counts from each regeneration library back to the reference using the Burrows-Wheeler Alignment Tool (BWA) [17] to produce a count table for each contig. Using DESeq version 1.12.1 [18] in R version 3.0.1 [19], samples were normalized for library size and their corresponding coefficient of biological variance was estimated. Differential expression (DE) was calculated based on negative*-*binomial distribution, selecting only the contigs that were significantly up- or down-regulated with an adjusted *P* value < 0.05.

**Fig. 3:**
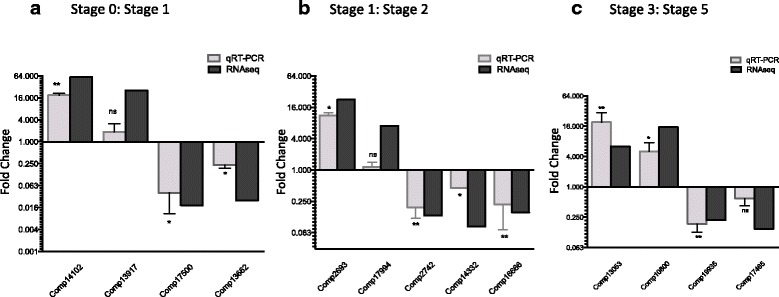
qRT-PCR validation of transcriptome DE analysis. **a** Shows the relative expression fold change for the four contigs that were significantly differentially expressed (adjusted *P* value <0.05) between Stages 0 and 1 in the RNA-seq data, next to their relative expression fold change for the qRT-PCR data (normalized against *RPL27* housekeeping gene). **b** Shows fold change of gene expression for five contigs that were significantly differentially expressed between Stages 1 and 2 in the RNA-seq data, next to the qRT-PCR gene expression. **c** Shows fold change of gene expression for four contigs that were significantly differentially expressed between Stages 3 and 5 in the RNA-seq next to the qRT-PCR gene expression. The contigs relative expression fold changes are given as the ratio of the first group compared to the second, (for example Stage 1 against Stage 0). The qRT-PCR expression data was analysed using a Mann–Whitney, non-parametric unpaired, two-tailed *t*-test, to determine if they were significantly differentially expressed, before converting them to fold change ratios

Each of the regeneration data sets used in the RNA-seq was validated technically and biologically using qRT-PCR on a subset of genes. This list was obtained by randomly selecting 13 differentially expressed transcripts from three pairwise comparisons: Stage 0–1, Stages 1–2 and Stages 3–5. qRT-PCR was performed on independently collected samples of RNA isolated from new biological replicates (Fig. 3). Each transcript resembles assembled reads making up the whole of part of a gene. A Mann–Whitney, non-parametric unpaired, two-tailed *t*-test was used to confirm if expression differences between WBR stages were significant. All qRT-PCR measurements were positively correlated with those from RNA-seq data. Furthermore, 10 of 13 transcripts evaluated by qRT-PCR were significantly differentially expressed (*P* value < 0.05) (Fig. 3).

To further confirm the correlation between RNA-seq and qRT-PCR data, the expression of six of our 13 contigs were compared between the vascular tissue (stage 0) and every subsequent stage of the WBR process (1–5). Expression fold changes (log2) were compared for RNA-seq and qRT-PCR data for each contig. All six contigs showed a high correlation between the RNA-seq and qRT-PCR data (Pearson coefficient correlation *r* = 0.8544, *R*^2^ = 0.7299, *P* value < 0.0001 (Additional file 5)). Overall the expression data was reproducible on independently collected biological samples. Taken together, these results confirmed the high accuracy of our RNA-seq analysis.

### Pathway analysis identifies biological processes expressed during WBR

We generated a list of significantly DE transcripts for early and late regeneration stages by pooling the transcriptomes corresponding to the first 24 h together (Stage 1 and 2), and the later stages, correlating to the next ~7 days together (Stages 3 and 4), and comparing each of these against the vascular-tunic tissue transcriptome (Stage 0). During early stages of WBR, we found 1853 contigs that were differentially expressed (adjusted *P* value < 0.05). Of these contigs, 811 were up-regulated and 1043 down-regulated in comparison to vascular-tunic tissue. In total, 287 unique *C. intestinalis* Ensembl IDs had a significant (E-value < 1e^−10^) match to one or more DE up-regulated contigs and 583 matches to DE down-regulated contigs. In the late WBR stages we identified 837 contigs (374 up-regulated and 463 down-regulated) that were differentially expressed (adjusted *P* value <0.05). We could find blast matches for 128 up-regulated and 181 down-regulated unique genes (adjusted *P* value < 0.05). Lists of differential expressed contigs were used to match to their closest counterpart in *C. intestinalis*. This was done to obtain differentially expressed genes (DEGs), to identify possible pathways important during the initial healing phase or later on in regeneration. We used KEGG pathway analysis and the PANTHER classification system [20, 21] in conjunction with these DEGs lists, to identify over-represented biological processes during early and later WBR.

### Early WBR requires both wound healing and known regeneration pathways

During the early stages of WBR (i.e. the first 24 h), twenty-seven biological processes were significantly overrepresented (*P* value < 0.05) in the DEGs (Additional file 6). These pathways included cell-cell (6 fold enrichment) and cell-matrix adhesion (12 fold enrichment) and other pathways important for the re-organization of tissue. This is to be expected, given that this is a critical period of closing and repairing the wound site. Apoptotic processes were 8-fold enriched, potentially initiating cellular clearance of damaged and dead tissue so that restructuring of the remaining vasculature can take place. A further 145 genes, encompassing primary metabolic and cellular processes (2 fold enrichment), reflect the high demand for energy required to fuel these repair and remodeling processes such as cellular proliferation. The last 18 genes pertain to signaling pathways with known involvement in developmental processes (3 fold enrichment). These biological processes are suggestive of a global switch in gene expression during the healing phase in *B. leachi*, as well as laying down the scaffolding for WBR to occur successfully during the later stages.

**Fig. 4:**
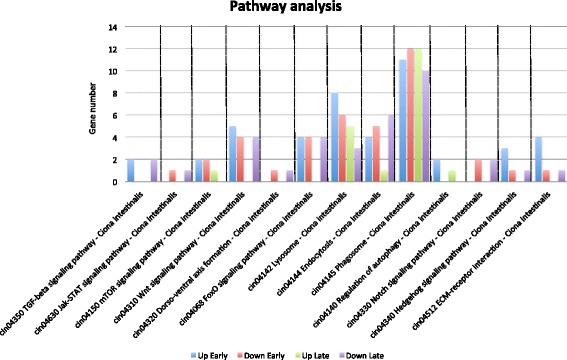
KEGG Pathway analysis. Graph of KEGG pathways analysis for those genes that were significantly differentially expressed (*P* value of 0.05). Weakly modulated components (not significant) within the pathways were not displayed. Note that all KEGG pathways can be found in Additional file 7

To add to our enrichment results and gain a broader vision of the involvement of the molecular players in the early stages of WBR, we performed a KEGG pathway analysis. Using this approach, we identified 106 pathways containing genes that were either up- or down-regulated during the first 24 h of WBR (Additional file 7). Specific pathways we identified as of interest to *B. leachi* regeneration included Wnt, Notch, TGF-β and Hedgehog signaling (Fig. 4). Importantly these pathways have previously been shown to be essential during chordate healing, regeneration and embryonic development [22–25], thus confirming the validity of our methodology.

**Fig. 5:**
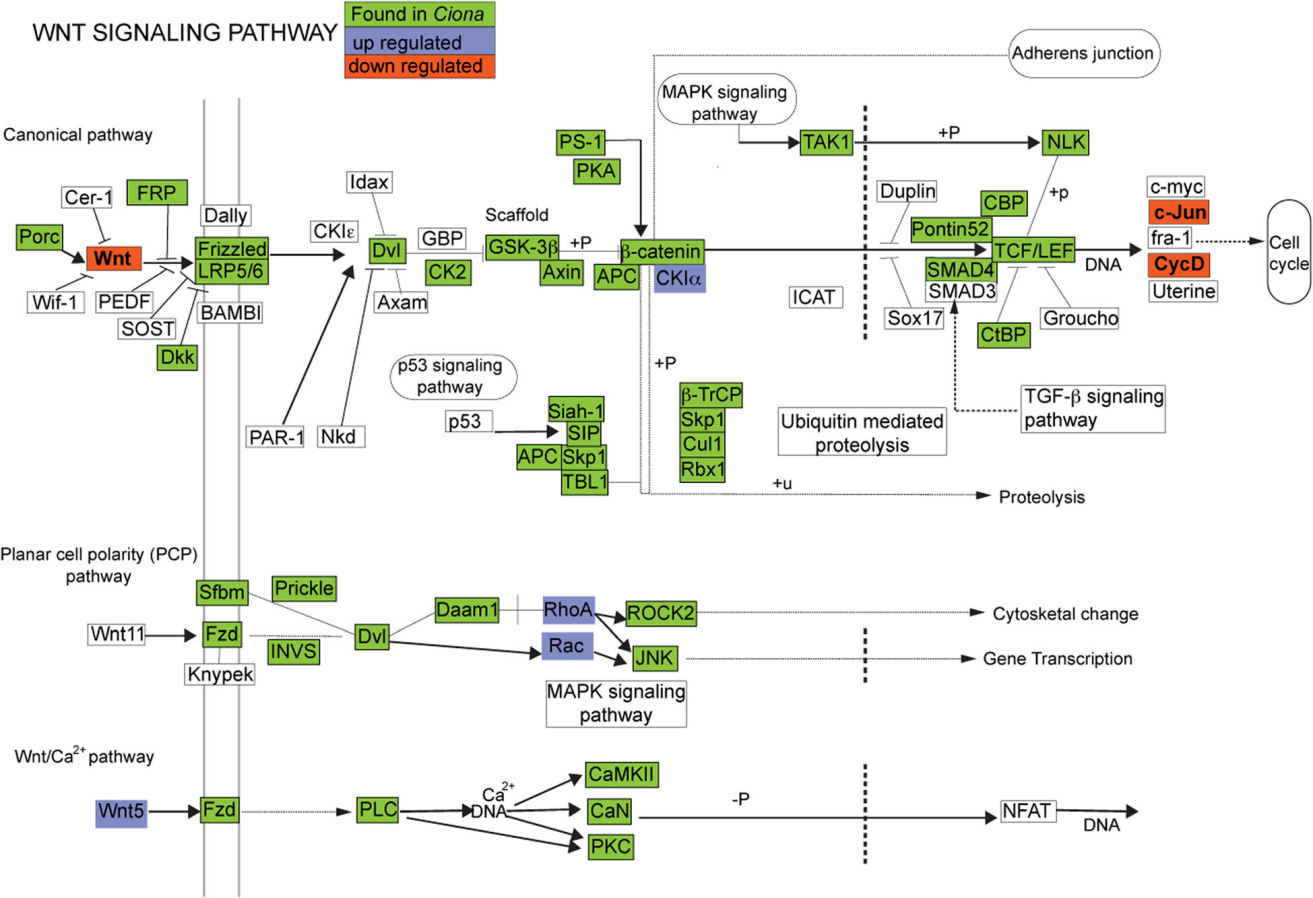
KEGG pathway map (cin04310) of the WNT Signaling Pathway with DE genes indicated. WNT pathway indicating contigs encoding components of the Wnt pathway significantly DE during early WBR (*P* value < 0.05). DE color code indicates genes that are up (blue) or down (pink) regulated. Image was modified from http://www.genome.jp/dbget-bin/www_bget?pathway+cin04310. Green-colored boxes indicate genes previously identified in the *C. intestinalis* genome (KEGG Mapper [56])

Of particular interest, all three Wnt pathways (canonical, planar cell polarity and calcium pathways [26–28] were highlighted by our analysis to contain DEGs (Fig. 5). Components of the Wnt pathway are up-regulated in many species undergoing healing and regeneration [29], including planarian [22], zebrafish [24] and newts [26]. Moreover, the Wnt pathway is activated shortly after injury and has been proposed to promote regeneration rather than mere healing [29, 30]. In our study, the canonical Wnt pathway was represented by a Wnt signaling ligand, *casein kinase 1 isoform alpha (CK1-alpha),* c-Jun and cyclin-D2-like (CycD), the non canonical planar cell polarity pathway by ras homolog gene family, member C (*rhoC*) and *rac1,* and the non canonical Wnt/calcium pathway by *Wnt5*. Up-regulation of CK1α a protein in the β-catenin destruction complex as well as repression of the Wnt ligand and downstream targets including c-Jun a transcription factor and CycD suggest that the Wnt canonical pathway is repressed. As for the planar cell polarity pathway, the measured increase in expression of *rhoC* and *rac1* could be associated with cell migration and polarity, presumably for the reorganization of the regenerating vascular epithelium. Lastly, the up-regulation of the non-canonical Wnt/calcium ligand by Wnt5 could suggest its involved in tissue regeneration, as Wnt5a has been shown previously to be required for colonic crypt regeneration in mice intestine [31], and in hydra during head regeneration [32]. On the other hand, *Wnt5b* expression in zebrafish was an inhibitor of the fin regeneration response [33]. Our data indicates the non-canonical and canonical Wnt signaling may play opposing roles in regulating the early regeneration response in *B. leachi*. However collectively, these pieces of data stress the involvement of Wnt ligands and associated components during early WBR, and their potential roles in repairing, remodeling and initiating the regeneration niche following amputation of the zooids.

Additionally, our analysis has emphasized the likely roles of components of the Notch signaling pathway in WBR, some of which have been shown previously to be active during wound healing and tissue repair in mammals [30, 34, 35]. In *B. leachi*, we detected a reduction in the expression of the receptor protein gene *Notch* (cin:723806) and its ligand, *Delta* (cin:778573) but an increase in a downstream transcriptional target *Hairy/Enhancer of split (Hes, e(spl)/hairy-b)* [30, 36]. *Hes* mediates the development of hematopoietic cells from stem cells precursors in mouse embryos [37]. Therefore, this pathway is of particular interest for further study, given that stem-like cells required for WBR are vascular-derived [8]. Furthermore, the down-regulation of both Notch receptor and ligand combined with the up-regulation of this downstream target suggests that the pathway may be briefly activated at the start of WBR (within the first few hours) and was being repressed when we captured RNA expression at 15/24 h.

Our analysis has also identified two groucho-like proteins (Gro) that may have roles in WBR. Gro is a co-repressor which interacts with DNA-binding proteins to silence gene transcription, facilitating the inhibition of multiple developmental signaling pathways including Wnt and Notch [35, 38]. Gro proteins are found only in metazoans and there are two known *groucho* genes in *C. intestinalis* [36]. In *B. leachi*, the differential expressed contig *Bl-groucho1* (comp35268_c0_seq1 ENSCINP00000017742) showed 3.1 fold down-regulation, while contig *Bl-groucho2* (comp13364_c0_seq2 ENSCINP00000017756) was up-regulated by 2.6 fold, early on during WBR.

Our analysis also detected the 1.9 fold up-regulation of contig comp17117_c0_seq2 which aligned to *TGF-β2 (*transforming growth factor beta superfamily signalling ligand, cin:778780). TGF-β2 is a cytokine with many essential roles during embryonic development including the regulation of cell differentiation, migration and apoptosis [39]. Importantly, the TGF-β pathway is required during regeneration of both the *Xenopus* tail [40] and the axolotl limb [41], and is also expressed early during WBR in *B. leachi* [1].

A further pattern of interest was the 2.3 fold up-regulation of contig comp15512_c0_seq1 which contained a sequence match to complement component C3-1 (cin:445694) in the KEGG pathway analysis. The complement pathway is a primordial pathway with origins tracing back to the earliest metazoans that is involved in innate immune response during regeneration [42]. In ascidians, *C. intestinalis* has been shown to have a complement system similar to the mammalian complement system in which C3-1 has many roles, including acting as a chemotaxin in hemocytes recruitment [43–45] and facilitating phagocytosis [46]. The up-regulation of this contig may indicate an immune response within the *B. leachi* colony following injury.

### Later stages of WBR correlates with increases in protein synthesis

During later WBR stages, there were a total of 10 biological processes that were significantly enriched (Additional file 6). Translational processes were by far the most prominent group (23 fold enrichment) and included regulation of translation and rRNA metabolic process. This reveals an increased requirement for protein synthesis during these later stages of regeneration. In addition, we found enrichment of genes associated with the biogenesis and the organisation of cellular components (11 and 3 fold respectively). All these molecular changes are consistent with the protein production, cellular proliferation and aggregation required for the creation of the opaque regeneration blastema (Fig. 1d-f).

Similarly to our analysis of early WBR, we performed KEGG pathway analysis on the later stages of WBR (results in Additional file 7). Of particular interest, we identified 58 genes up-regulated in the ribosomal pathway. The large increase in protein production strongly suggests a greater demand in cell proliferation needed for the newly developing zooid to take shape. Moreover, regeneration has been shown to require proliferation of *Piwi +* cells between days two and ten, post zooid removal [3, 8]. Together with the transcriptome data, our analysis demonstrates the massive increase in cell proliferation that is critical for WBR.

Given the central role of the Wnt, Notch and TGF-β pathways during the early stages of WBR, we examined how they were modulated later on. Interestingly, genes up-regulated in the Wnt pathway in early regeneration, both in the canonical and non-canonical pathways, declined in later stages. Similarly, bmp2/4 cin:778554 in the TGF-beta showed down-regulation. On the contrary, Notch components were down-regulated during both the early and the later phases of WBR. This raises the possibility that up-regulation of Notch expression is required only transiently shortly after injury. Given that both Wnt and Notch pathways are required for angiogenesis [47, 48], these results reflect the global transcriptional shift from healing and remodeling vascular tissues within the tunic to creating regeneration niches and developing the regeneration niche.

## Conclusion

Whole body regeneration in colonial ascidians is the most dramatic example of regeneration in the chordates phylum, and thus the evolutionarily closest illustration of complete regeneration to the limited extents present in vertebrates. Despite this unique advantageous position, very little is currently known about the molecular pathways underlying WBR in botryllid species. Using RNA-seq, we performed the first unbiased study of gene expression dynamics across regeneration in *B. leachi*. In addition to providing ample research material for further investigation on the transcriptome during WBR, our current analysis has detected genes and signaling pathways previously identified in *B. leachi*, include *Notch/Delta*, *JAK/STAT, Ras* and *TGF-β* [1, 3, 6]. Moreover, our *de novo* transcriptome also detected previously uncharacterized molecular players. Several known developmental pathways such as Wnt and TGF-β were up-regulated as part of the wound healing and vasculogenesis response, which occurs within the first 24 h following zooid amputation. Many of the up-regulated pathways have evolutionarily conserved roles in stem cell generation, proliferation, differentiation and patterning of tissues [1, 49–51]. The dynamic modulation of their molecular components therefore appears both legitimate and essential during *B. leachi* WBR. However given the complex nature of these pathways, further functional analysis will need to be carried out to dissect their involvement in the regeneration process.

Interestingly, we observed a distinctive change in gene expression pattern over the course of WBR with early genes important for healing and later ones for protein synthesis, cellular component biogenesis and organization. Importantly, many regeneration studies have shown that there is an initial healing phase following injury before regeneration can proceed. The epithelial layer, that closes the wound site, is thought to generally be a likely source of signaling for regeneration [49]. In *B. leachi* molecular studies, and histological studies (manuscript in preparation) have shown there is an initial healing phase as well. However the induction signal is unlikely to come from the reestablished epithelial lining as WBR, rather than healing, only occurs when all zooids from the colony are lost. If any adults remain attached to the vascular tissue the colony will merely undergo wound healing by closing of the wound site and reorganising the vascular system around the remaining zooids [3]. It will thus be important to identify the genes differentially expressed between this healing process and entry into WBR to identify how those molecular programs are triggered.

This study provides the first unbiased analysis of the drastic gene expression changes occurring during the deeply dynamic process of whole body regeneration in a chordate model. Our work provides a basis for future studies identifying genes and pathways important during *B. leachi* WBR.

## Methods

### *B. leachi* collection and husbandry

*B. leachi* colonies were collected from both the Otago harbour (latitude 45.87°S, longitude 170.53°E) and Nelson harbour (latitude 41.26°S, longitude 173.28°E) in New Zealand. *B. leachi* grows naturally on submerged structures (e.g. rocks, ropes, pontoons, tires) and colonies were removed from the attached substrates with a razor blade. Each individual colony was placed on 5 × 7.5 cm glass slides and left horizontally for two days in still water to allow the colony to attach to the slide. The slides were then placed vertically and kept in a tank of 17 °C–20 °C aerated salt water. The glass slides were cleaned of contaminating organisms by using a paintbrush and tungsten needle as per [6].

### *B. leachi* regeneration

*B. leachi* regeneration was carried out following established protocol [6]. Dissection of *B. leachi* marginal ampullae was carried out using a fine tungsten needle. The blood vessel fragments attached to the slides were returned to the aerated saltwater tanks and left to regenerate until a certain morphological stage of regeneration had been reached. Regenerating tissues were then removed from the glass slides for RNA isolation.

### RNA extraction

RNA was extracted from 2–6 individual *B. leachi* fragments and samples were pooled together to allow enough tissue to be present for RNA extraction. The Spectrum™ Plant Total RNA Kit (Sigma) was used on fresh tissue as per manufactures instructions to isolate RNA from pooled samples. The RNA quantity was checked on a Qubit 2.0 Flurometer and its quality on a Bioanalyzer (Agilent). Quality checks indicated that the total RNA quality was excellent, with samples possessing a *RNA* integrity number (RIN) of 9.9 or 10, well above the recommended RIN of >7.

### Pre-assembly checks, raw data quality and processing

Sequencing was carried out at New Zealand Genomics Limited (NZGL) using Illumina HiSeq 2000 platform. RNA sample libraries were prepared using Illumina TruSeq™ standard mRNA Library Preparation kit. Fragments >200 bp were selected for the final enriched libraries with an average insert size of 324 +/− 7 bp across all libraries. Paired-end sequencing of 2× 100 bp was carried out generating approximately 34 million reads per lane. The raw sequence data was provided in FASTQ format. The raw reads were checked with FASTQC (http://www.bioinformatics.babraham.ac.uk/projects/fastqc/) for quality control. Reads were subsequently filtered to fit Q30 for the full length of a read. Quality trimming and length sorting were performed using DynamicTrim.pl and LengthSort.pl, (http://solexaqa.sourceforge.net). The *de novo* transcriptome was assembled using Trinity (version trinityrnaseq_r2013_08_14) using default software settings [10]. A reference transcriptome was assembled using a subset of reads from all eight libraries sequenced (regeneration stages 0–5, whole colony and embryo). Reads from each of the regenerative stages were mapped back to the reference transcriptome using the Burrows-Wheeler Alignment Tool (BWA) [17] to produce a count table for each contig at every sequenced stage. A pairwise comparison using the PlotMDS function in edgeR [52] was used to produce the MDS plot (method = bcv, top = 500). This function uses the top 500 genes with the largest biological variation between samples (pairs), based on the average across the whole population.

### qRT-PCR

Total RNA was extracted from independent biological replicates for each stage of *B. leachi* regeneration using Sigma Spectrum Plant Total RNA Kit. RNA was DNase treated before using reverse transcription to make cDNA. All samples were tested in triplicate with SYBR Master Mix (Life Technologies). qRT-PCR data was analysed with the ΔCt method using 2^-(ΔCt)^ to work out relative RNA expression changes for the contigs [53] to the housekeeping gene *RPL27* [54]. Fold change was then worked out against vascular tissue using the 2^-(ΔΔCt)53^. A melt-curve analysis was also performed to confirm the production of a single amplicon for each set of oligonucleotide primers. A list of qPCR primers is provided in Additional file 8 including their corresponding efficiency.

### Biological process analysis

PANTHER (http://pantherdb.org/) was used to carry out biological process analysis on the significantly differentially expressed genes identified in the early and late stages of *B. leachi* regeneration. Only up-regulated contigs with a significant (E-value cut off at < 1e-10) to *C. intestinalis* protein matches were used. An over-representation test was carried out to identify biological processes that had more genes expressed than background levels. The PANTHER GO-slim biological process function was used which applies a Bonferroni correction to account for multiple testing. *Ciona intestinalis* was used in PANTHER as the background species.

### KEGG pathway analysis

The differentially expressed Ensembl ID lists corresponding to Early and Late stages of WBR were converted to Entrez IDs using BioMart [55] for use in KEGG pathway analysis. A p-value of 0.05 was used to identify a maximum number of DE contigs within each pathway. The “Search & Color Pathway” tool (http://www.genome.jp/kegg/tool/map_pathway2.html) using *Ciona intestinalis* as the background.

### Availability of supporting data

The raw sequencing reads supporting the results of this article are available in the NCBI Sequence Read Archive (SRA; http://www.ncbi.nlm.nih.gov/sra) repository, under accession numbers SRP064769.

## Additional files

Additional file 1:
**RNA sequencing statistics and quality.** (PDF 21 kb) Additional file 2:
**Core eukaryotic gene (CEGs) completeness statistics.** (PDF 29 kb) Additional file 3:
***De novo*** **assembled** ***B. leachi*** **transcripts with significant match to the** ***C. intestinalis*** **protein database (E-value cut off at < 1e-10).** (XLSX 1950 kb) Additional file 4:
***De novo*** **assembled** ***B. leachi*** **transcripts with significant match to the UniProt protein database (E-value cut off at < 1e-10).** (XLSX 2325 kb) Additional file 5:
**Correlation between RNA-seq and qRT-PCR data.** (PDF 39 kb) Additional file 6:
**PANTHER overrepresentation test showing the GO-Slim Biological Process that were overrepresented among the DE contigs in both early and later stages of WBR.** (XLSX 48 kb) Additional file 7:
**KEGG Pathway analysis with DEG during early and late WBR, in comparison to Stage 0.** (XLSX 13 kb) Additional file 8:
**List of qPCR primer sequences with their corresponding efficiency.** (PDF 57 kb)

## Abbreviations

bp: base pairs
DE: differential expression
DEGs: differentially expressed genes
h: hours
min: minutes
WBR: whole body regeneration
