# Supplementary material for: Uncovering the pathways underlying whole body regeneration in a chordate model, Botrylloides leachi using de novo transcriptome analysis
Source: BMC Genomics. 2016 Feb 16;17:114. doi: 10.1186/s12864-016-2435-6 (PMC4755014; doi:10.1186/s12864-016-2435-6)
Supplement: Additional file 1: — RNA sequencing statistics and quality. (PDF 21 kb) [file 12864_2016_2435_MOESM1_ESM.pdf]

Sequencing ID	Total reads	%data>Q30	Ave Qscore
Embryos (S:E)	40,691,968	90.43	35.33
Adult (S:A)	44,002,486	91.83	35.74
Stage 0 (S:0)	40,836,770	91.5	35.66
Stage 1 (S:1)	39,180,842	91.71	35.72
Stage 2 (S:2)	40,081,420	91.6	35.69
Stage 3 (S:3)	39,353,946	91.3	35.59
Stage 4 (S:4)	33,640,776	91.37	35.61
Stage 5 (S:5)	37,800,008	91.06	35.5
Total Transcriptome	315,588,216		
