# Supplementary material for: Uncovering the pathways underlying whole body regeneration in a chordate model, Botrylloides leachi using de novo transcriptome analysis
Source: BMC Genomics. 2016 Feb 16;17:114. doi: 10.1186/s12864-016-2435-6 (PMC4755014; doi:10.1186/s12864-016-2435-6)
Supplement: Additional file 2: — Core eukaryotic gene (CEGs) completeness statistics. (PDF 29 kb) [file 12864_2016_2435_MOESM2_ESM.pdf]

Transcriptome (Trinity)	Number of 248 ultra-conserved CEGs present in genome	Percentage of 248 ultra-conserved CEGs present	Group 1%	Group 2%	Group 3%	Group 4%
**Complete**	237	95.56	90.91	94.64	100.00	96.92
**Partial**	244	98.39	96.97	96.43	100.00	100.00
