# Supplementary figures and images for: Uncovering the pathways underlying whole body regeneration in a chordate model, Botrylloides leachi using de novo transcriptome analysis
Source: BMC Genomics. 2016 Feb 16;17:114. doi: 10.1186/s12864-016-2435-6 (PMC4755014; doi:10.1186/s12864-016-2435-6)

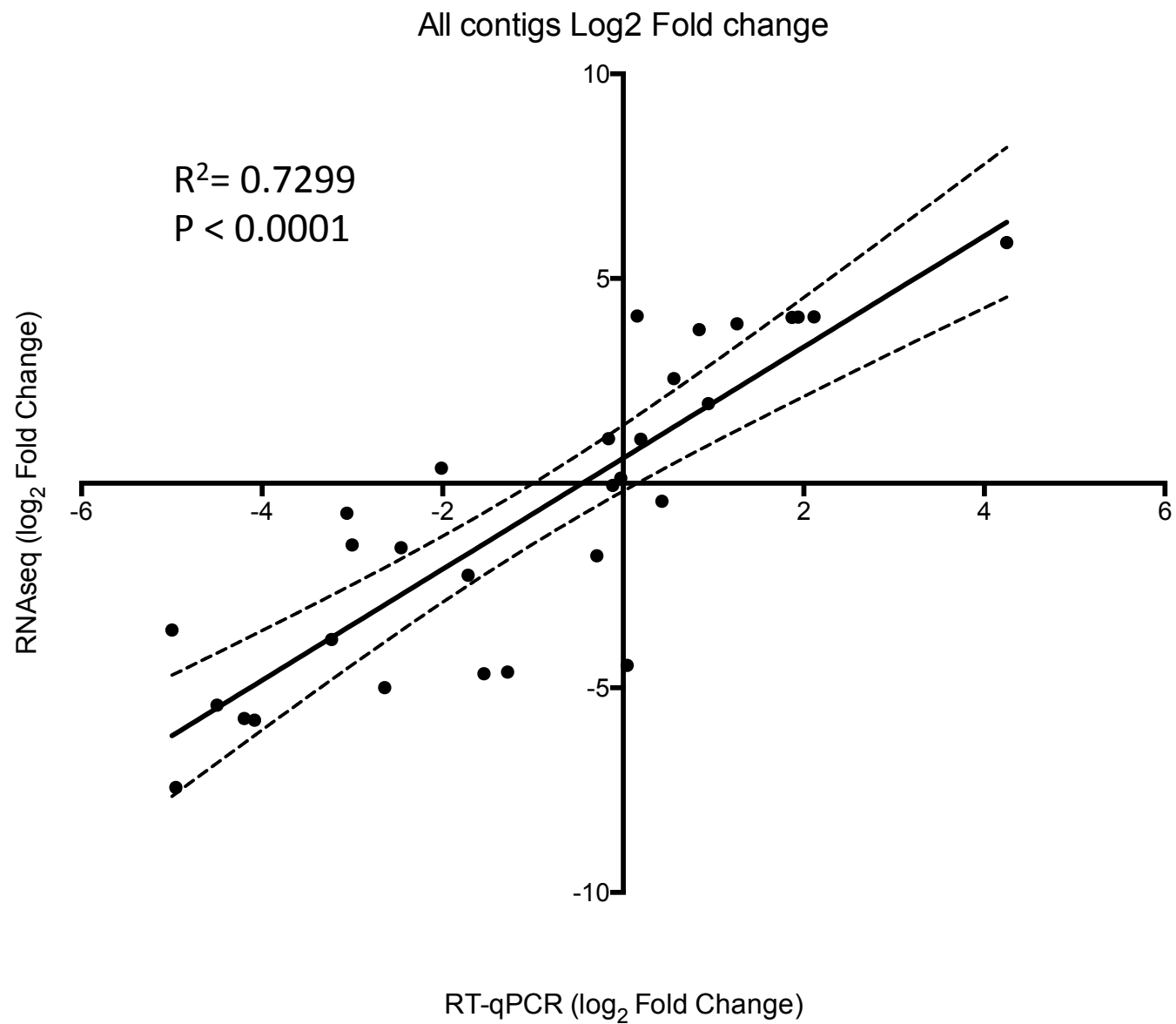

Supplement: Additional file 5: — Correlation between RNA-seq and qRT-PCR data. (PDF 39 kb) [file 12864_2016_2435_MOESM5_ESM.pdf]
