# Supplementary material for: Uncovering the pathways underlying whole body regeneration in a chordate model, Botrylloides leachi using de novo transcriptome analysis
Source: BMC Genomics. 2016 Feb 16;17:114. doi: 10.1186/s12864-016-2435-6 (PMC4755014; doi:10.1186/s12864-016-2435-6)
Supplement: Additional file 8: — List of qPCR primer sequences with their corresponding efficiency. (PDF 57 kb) [file 12864_2016_2435_MOESM8_ESM.pdf]

RT-qPCR primer information

Contig id	Oligo Sequence (Forward)	Oligo Sequence (Reverse)	Amplicon Length (bp)	Primer Efficiency %
**Stage 0-1**				
**comp14102_c0_seq1**	CTTCCTACCACAGCTCAGAAAC	ACCATTGACCGATTGCACTAT	111	102.4%
**comp13917_c0_seq1**	TCCTGATGTTGTGCCGTATTC	CAAGGTCACAGGAGTTGTAAGT	97	103.2%
**comp17500_c0_seq1**	CGCATCAATCAGTCGGGATAA	GACTTCTTGTCCTCTCCCAAAG	99	98.7%
**comp13662_c1_seq1**	AACGAGGAACTTGAGACCAATC	GGCACTTCTGTCCTGTCATATC	83	103.8%
**Stage 1-2**				
**comp2693_c0_seq1**	GGGAAGATGGTGACCGTTATT	AGGATGGTTGCCATGTGTAG	86	96.3%
**comp17994_c0_seq1**	CCGAAAGTGTTGTGGCAATATC	CCAAAGGTGTAGCTCTGGTTAT	99	99.8%
**comp2742_c0_seq1**	CCTACGTAGCATTGCCTGTATC	GTGCGACCTTCATATCCGTATC	143	95.2%
**comp14332_c0_seq1**	CAGTGACGGTGATGGAGTATG	GCGAATGAACCAGGAGTAAGA	120	96.3%
**comp16666_c0_seq1**	GACCTAGATGTCTCGTTTCGTTAG	GATCTGTCTGACTGTGGTGAAG	84	94.9%
**Stage 3-5**				
**comp13053_c0_seq1**	GTACTCTTCTCGCGGAATTGAT	ACATACCTTCACCCTTGGATTG	99	101.2%
**comp10800_c0_seq1**	GTCAATCCCATCTCCTGAGTTT	CATGGTTGGAGAAGCTCTTGTA	97	104.4%
**comp19935_c0_seq1**	CATACGCGACTCGTGGTAATA	CAGAGATGGAATGTGGTTGATTG	107	96.1%
**comp17465_c0_seq1**	GGTTGAAGGCAGCAATGTAATC	GGTAGGTCCTCTTCACTTCTTG	101	94.7%
